# Role of self-management program based on 5A nursing model in quality of life among patients undergoing hemodialysis: a Randomized Clinical Trial

**DOI:** 10.1186/s12882-023-03108-2

**Published:** 2023-03-15

**Authors:** Sahar Keivan, Abdolali Shariati, Mojtaba Miladinia, Mohammad Hosein Haghighizadeh

**Affiliations:** 1 Department of Nursing, School of Nursing and Midwifery, Ahvaz Jundishapur University of Medical Sciences, Ahvaz, Iran; 2 Nursing Care Research Center in Chronic Diseases, School of Nursing and Midwifery, Ahvaz Jundishapur University of Medical Sciences, Ahvaz, Iran; 3 Department of Biostatistics, School of Health, Ahvaz Jundishapur University of Medical Sciences, Ahvaz, Iran

**Keywords:** Self-management, Self-care efficacy, Hemodialysis, 5A nursing intervention, Quality of life

## Abstract

**Introduction:**

Various nursing models are usually employed to achieve self-management and improve the quality of life in chronic conditions. Given its person-based characteristics, the 5 A nursing model can improve the quality of life of hemodialysis patients.

**Purpose:**

This study aimed to determine the role of a self-management program based on the 5 A nursing model in the quality of life of patients undergoing hemodialysis.

**Materials and methods:**

This clinical trial was conducted on hemodialysis patients in Iran. Random sampling was adopted to assign 60 patients to intervention and control groups. After the participants completed a demographic questionnaire and the *Kidney Disease Quality of Life–Short Form (KDQOL–SF)*, routine measures were taken in the control group. However, the 5 A nursing model was implemented in the intervention group for three months. The self-care program was executed in face-to-face sessions or via phone calls and SMSs. After three months, the quality of life was measured again in both groups.

**Findings:**

There were significant differences after the intervention between the intervention and control groups in specific dimensions of quality of life, such as cognitive functions, symptoms, sleep, dialysis, social support, and renal complications (P < 0.05). The two groups also had significant differences in the general scores of quality of life (P < 0.05).

**Conclusion:**

The 5 A self-management intervention as a person-based model could improve self-care in hemodialysis patients. Nurses can implement this model to mitigate care costs, enhance interventions, and improve patients’ quality of life.

**Trial registration:**

Iranian Registry of Clinical Trials (IRCT20211103052955N1; 19/11/2021).

## Introduction

Chronic kidney disease is a public health threat worldwide. The global prevalence of chronic kidney failure is 262 cases per one million. Hemodialysis patients experience various side effects such as insomnia, skin irritation, headaches, blood pressure disorders, vascular complications, muscle cramps, itches, nausea, and vomiting, which can affect different dimensions of their quality of life [1, 2]. Although many developments have been made in hemodialysis, this complicated process requires a caregiver team and many instructions to improve the quality of life among patients with hemodialysis [3]. Since the quality of life is affected in patients with hemodialysis, appropriate care methods should be adopted to mitigate the effects through lifestyle moderation. Hence, self-management interventions can be instrumental tools to support the necessary lifestyle changes in hemodialysis [4].

The self-management program is a rehabilitation method in which patients play a critical role. All healthcare activities focus on patients to achieve self-decision-making, maximize independence, and improve personal health based on abilities and lifestyles by enhancing the quality of life [5]. Self-management refers to personal abilities to control symptoms, physical outcomes, treatments, and social-psychological effects of chronic cases such as dialysis patients who need to control their lifestyle changes [5, 6]. Studies have shown that self-management interventions such as training patients and using care models can effectively improve disease symptoms and patients’ quality of life, especially regarding compliance with medical prescriptions, functional instructions, and patient satisfaction [7].

Different nursing models are usually employed to achieve self-management and improve the quality of life in chronic cases [8]. As a behavioral modification model, the 5 A nursing model is an evidence-based approach designed to change behavior and achieve self-management. Presented by Glasgow et al. (2003), this model offers nursing intervention through assessment, advice, agreement, assistance, and arrangement stages. In fact, this model provides a valuable framework for executing self-management interventions [9]. This model has improved care outcomes in some chronic cases; however, no improvements were reported in some settings [10–12].

The chronic nature of renal failure and dependence on hemodialysis for survival can impose high costs on patients and decline the quality of their lives. Thus, patients worry about their abilities to do daily tasks and live normally. Due to these patients’ various complications, comprehensive nursing interventions are essential, emphasizing rehabilitation programs. Therefore, necessary arrangements should be made to allow patients to take responsibility for improving self-efficacy and enhancing self-management behaviors. There are weaknesses in conventional education strategies. At the same time, the patient’s active participation in the therapeutic process and hemodialysis is essential. On the other hand, although the 5 A model has been assessed for the quality of life of patients with acute coronary syndrome, hypertension, and diabetes patients, the researcher did not find any study investigating the effect of the self-management program based on the 5 A model on the quality of life of hemodialysis patients. Hence, this study aimed to determine the role of a self-management program based on the 5 A nursing model in the quality of life of hemodialysis patients.

## Methods and materials

### Design and setting

This parallel-group randomized clinical trial was conducted in the Dialysis Center of Imam Ali Hospital, affiliated with Ahvaz Jundishapur University of Medical Sciences in Khuzestan Province, Iran, in 2021–2022 (Iranian Registry of Clinical Trials; IRCT20211103052955N1; 19/11/2021).

### Participants

**Fig. 1:**
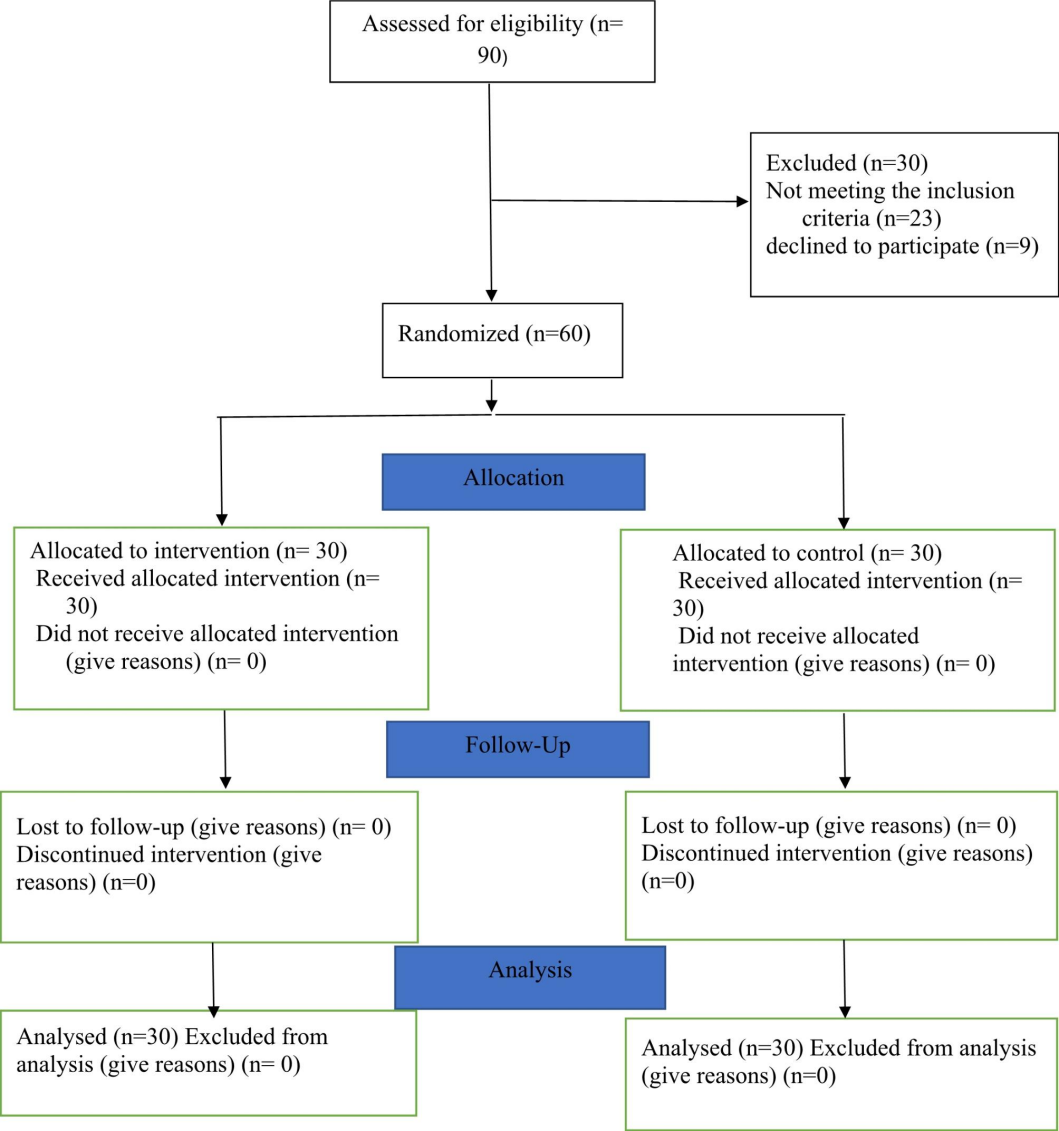
CONSORT flowchart

In this trial, the convenience sampling method was adopted to select 60 patients undergoing hemodialysis. The inclusion criteria were literacy, no history of psychological disorders, age of 18 to 65 years old, and no history of cognitive disorders such as Alzheimer’s disease. The participants were randomly assigned to intervention and control groups with 30 members each. The permuted block randomization technique was employed to allocate the participants. The blocks were randomly determined as 2, 4, 6, and 8, whereas a statistician prepared the randomization list. The sampling attrition rate was considered 10%. Based on a similar study [13] and the following formula, 30 participants were allocated to each group with a 95% confidence interval (CI) and a test power of 80%. Moreover, participants were free to leave the study in case of unwillingness or the emergence of serious problems (Fig. 1). The principal investigator and statistician were blinded.

$${\times }_{1}=11/5$$ $${\times }_{2}=9/67$$ $${\delta }_{1}=2/12$$ $${\delta }_{2}=2/6$$

$$n = \frac{{\left( {\sigma _1^2 + \sigma _2^2} \right){{\left[ {{z_{1 - \alpha /2}} + {z_{1 - \beta }}} \right]}^2}}}{{{{\left( {{M_1} - {M_2}} \right)}^2}}}$$

### Intervention

**Fig. 2:**
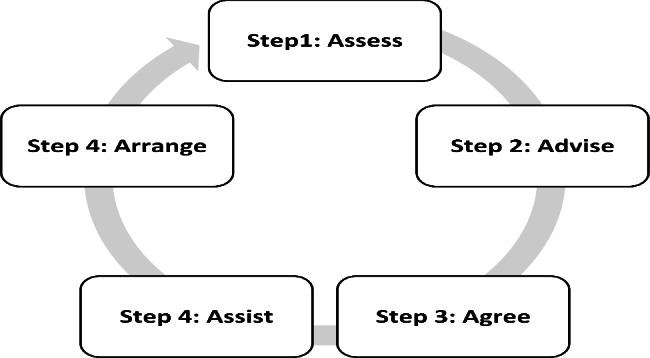
The 5 A model algorithm

The intervention group received a self-management program based on the 5 A model, which was implemented in five stages through face-to-face meetings, phone calls, and SMSs in three months. Figure 2; Table 1 present the 5 A model steps and their implementation methods in detail [13]. Moreover, the control group received the routine hospital program, including conventional care and training measures.

**Table 1:** The 5 A Model Steps in Hemodialysis Patients

Step 1	Assess	In this step, patients were analyzed in face-to-face interviews regarding risk factors, history of diseases, renal complications, compliance with pharmaceutical prescription, sleeping status, nutrition, type of activity, and case information.
**Step 2**	Advise	In this step, the previous analysis results were considered to inform patients of the diagnosed health risks and emphasize the benefits of behavioral modification.
**Step 3**	Agree	An agreement was reached between the patients and the researcher. Given the diagnosed problems, appropriate behavioral goals were agreed upon with patients, and a practical program was designed for each goal. The criterial need was set between 0 and 1 for each behavioral goal so that patients could determine their trust in the program implementation. These criteria were registered in the behavioral modification form, and the patients were asked to record their performance status in each behavioral goal weekly for 12 weeks.
**Step 4**	Assist	The patients were instructed on how to control the consumption of liquids, how to implement care measures through vascular access, the importance of sports and necessary levels of physical activities, how to take care of their skin, and compliance with their diets and prescriptions in face-to-face sessions. The patients were asked to perform the sports exercises daily and then record their performance results. Proportionate to the needs of patients, a personal training session was arranged respecting the patient’s willingness (in-person or via phone calls) to reiterate and emphasize the instructions.
**Step 5**	Arrange	In this step, the functions of patients were followed-up for three months. In fact, they were followed up via phone calls or SMSs three times a week from the fifth week to the 12th week every week to ensure that the patients complied with the intervention within the first four weeks. These efforts were made to remind patients of the practical program and solve any potential problems. Furthermore, each patient’s progress was followed up every four weeks in one face-to-face session to modify goals or useful programs by reaching new agreements or encouraging patients to keep up the intervention.

### Data Collection

A three-section tool was employed for data collection. The first section gathered data on clinical and demographic variables such as age, gender, educational attainment, and hemodialysis treatment duration. The second section comprised the *Kidney Disease Quality of Life–Short Form (KDQOL–SF)*. In the third section, a checklist of the 5 A model steps was employed to only record the 5 A model steps within a unified framework. The *KDQOL–SF* is a self-administered questionnaire consisting of general and specific dimensions regarding the quality of life, created by Hayes et al. in 1994. The general dimension of quality of life included two other dimensions (i.e., physical health and psychological health) and eight areas. The physical dimension included four areas: general health, physical performance, physical role, and physical pain, whereas the psychological dimension comprised emotional role, social performance, psychological health, and happiness. Moreover, the specific dimension had 11 areas, including symptoms and problems, effects of renal diseases, burden of renal diseases, cognitive performance, quality of social relationships, social support, sleeping status, employment status, sexual problems, and satisfaction with care and personnel. This multidimensional tool is reliable and valid, including all dimensions of the SF–36 Questionnaire plus the kidney disease variables. It also has high internal consistency and correlation. Each dimension is scored from 0 to 100. Scores above 50 on each dimension and area indicate good quality of life. The reliability and validity of the Persian version of *KDQOL–SF* were confirmed by Yekaninejad et al. in Iran (α = 0.77–0.9) [14]. This questionnaire was completed before the intervention and three months after the intervention.

### Data Analysis

Data analysis was performed in SPSS 18 at a significance level of 0.05 using the independent *t*-test, paired *t*-test, and ANOVA. However, nonparametric tests were conducted in case of a non-normal distribution or a ordinal qualitative variable.

## Results

The age variable is expressed separately as mean and standard deviation for the control and intervention groups. The average age was 52.83 ± 8.71 years in the control group and 52.66 ± 9.23 years in the intervention group, without a statistically significant difference (P = 0.943).

The findings indicated that most patients were male (56%), married (78%), and urban residents (98%). There were no significant differences between the two groups regarding demographic variables such as age, marital status, place of residence, educational attainment, and history of renal transplants (P > 0.05). However, the two groups differed significantly in terms of some demographic features, such as employment status (P = 0.009), economic status (P = 0.011), and the number of dialysis sessions (P = 0.001) (Table 2). The distortive effects of these variables were analyzed through ANCOVA (P > 0.05); hence, it could be concluded that the intervention caused intergroup differences and that the abovementioned factors did not affect the results.

**Table 2:** Distribution of frequency and percentage of demographic characteristics of patients in intervention and control groups

variable	Classification	intervention group	control group	p-value
		number (percentage)	number (percentage)	
**Gander**	man	(53.3)16	60.0))18	0.602
	woman	(46.7)14	(40.0)12	
**Marriage status**	single	(3.3)1	(13.3)4	0.369
	married	(83.3)25	(73.3)22	
	divorced	(13.3)4	(13.3)4	
**Address**	city	(96.7)29	(100.0)30	0.313
	village	(3.3)1	(0.0)0	
**Education**	Literacy for reading and writing	(53.3)16	(36.7)11	0.189
	Under diploma	(30.0)9	(20.0)6	
	Diploma	(13.3)4	(13.3)4	
	university	(3.3)1	(0)0	
**Job status**	free	(70.0)21	(36.7)11	0.009
	freelance job	(26.7)8	(26.7)8	
	Government	(0.0)0	7(13.3)	
	retired	(3.3)1	(23.3)7	
**Economic status**	Income equals expenses	(28.6)25	(50.0)15	0.011
	More income than expenses	1(3.4)	(6.7)2	
	Income less than expenses	3(10.3)	(43.3)13	
**Number of dialysis sessions**	2 times a week	23(76.7)	(40.0)24	0.001
	3 times a week	7(23.3)	(60.0)36	
**Kidney transplant history**	no	(14.3)4	(10.3)3	0.650
	yes	(85.7)24	(89.7)26	

According to the results, there were significant differences between the two groups before the intervention concerning the scores of some specific dimensions of quality of life, such as renal complications, dialysis, patient satisfaction, and quality of social interactions. The distortive effects of these variables were analyzed through ANCOVA. Given the P-value (P = 0.001), it could be concluded that the intervention caused intergroup differences, and the abovementioned factors did not affect the results. The independent *t*-test indicated no significant differences between the two groups after the intervention regarding the specific dimensions of quality of life, such as sexual performance, employment status, quality of social interactions, and patient satisfaction. However, the two groups significantly differed in other dimensions, such as cognitive performance, symptoms, sleeping status, dialysis, social support, and renal complications (Table 3).

**Table 3:** Intergroup comparison of specific aspects of quality of life of patients before and after intervention in intervention and control groups

variable	Intervention group		Control group		**p-value	*p-value
	Before intervention	After intervention	Before intervention	After intervention		
	mean ± standard deviation	mean ± standard deviation	mean ± standard deviation	mean ± standard deviation		
**Symptom .problem**	58.26 ± 14.39	72.36 ± 10.67	59.37 ± 18.79	60.06 ± 19.49	0.004	0.798
**Effect of kidney disease**	9.58 ± 17.34	42.50 ± 13.37	28.12 ± 26.40	27.29 ± 25.61	0.006	0.002
**Work status**	5.00 ± 20.12	5.55 ± 21.18	13.79 ± 32.44	14.28 ± 32.93	0.249	0.214
**Cognitive function**	51.33 ± 22.36	72.66 ± 14.89	58.44 ± 25.94	57.11 ± 23.13	0.003	0.260
**Quality of social interaction**	46.44 ± 20.52	70.44 ± 14.61	61.77 ± 25.48	61.66 ± 25.58	0.154	0.013
**Sexual function**	17.91 ± 18.47	29.16 ± 23.29	31.94 ± 33.13	30.60 ± 32.14	0.844	0.050
**Sleep**	46.83 ± 15.38	68.58 ± 12.38	50.25 ± 12.66	49.41 ± 12.53	0.001	0.352
**Social support**	79.88 ± 25.73	94.44 ± 11.01	71.10 ± 25.87	66.66 ± 25.89	0.001	0.197
**Dialysis staff encouragement**	90.83 ± 17.34	98.33 ± 7.14	66.37 ± 29.71	63.75 ± 25.71	0.001	0.001
**Patient satisfaction**	36.66 ± 16.02	62.21 ± 13.08	59.44 ± 22.60	56.66 ± 23.81	0.001	0.001

*p-value- Before intervention

**p-value After intervention

Concerning the general dimensions of quality of life before the intervention, there were significant differences between the two groups regarding some dimensions, such as energy level, emotions and feelings, role limitation due to emotional and physical problems, and general health. The distortive effects of these variables were analyzed through ANCOVA, which indicated that the intervention caused intergroup differences and that the above mentioned factors did not affect the results. According to the independent *t*-test, there were no significant differences between the two groups after the intervention regarding the general dimensions of quality of life, such as role limitation due to emotional and physical problems, pain, and general health. However, the two groups differed significantly in other general scales of quality of life, such as physical performance, emotions and feelings, social performance, general health status, and energy level. The intragroup comparison of the general dimensions of quality of life indicated that all dimensions in both groups changed significantly after the intervention (Table 4).

**Table 4:** Between-group and intra-group comparison of the general dimensions of patients’ quality of life before and after the intervention in the intervention and control groups

variable	Intervention group		Control group		**p-value	*p-value
	Before intervention	After intervention	Before intervention	After intervention		
	mean ± standard deviation	mean ± standard deviation	mean ± standard deviation	mean ± standard deviation		
**Physical function**	55.50 ± 16.88	72.33 ± 19.46	55.66 ± 28.00	54.33 ± 28.09	0.978	0.978
p-value ***	0.001		0.001			
**Role limitation due to physical problems**	90.83 ± 26.65	61.66 ± 24.33	65.83 ± 39.10	64.65 ± 37.51	0.005	0.005
p-value ***	0.001		0.001			
**Pain**	34.33 ± 22.28	51.66 ± 20.98	41.41 ± 26.38	41.00 ± 25.24	0.266	0.266
p-value ***	0.001		0.001			
**General health**	14.00 ± 17.24	34.16 ± 20.43	38.83 ± 22.46	37.00 ± 21.99	0.001	0.001
p-Value ***	0.001		0.001			
**Emotional well-being**	39.73 ± 17.79	68.53 ± 8.25	53.73 ± 18.45	55.33 ± 19.51	0.004	0.004
p-value ***	0.001		0.001			
**Role limitation due to emotional problems**	91.11 ± 23.04	44.44 ± 23.70	58.88 ± 39.81	59.52 ± 38.86	0.001	0.001
p-value ***	0.009		0.009			
**Social function**	47.50 ± 17.49	65.41 ± 12.14	48.33 ± 22.19	49.16 ± 22.72	0.872	0.872
p-value ***	0.001		0.001			
**Energy-Fatigue**	27.50 ± 21.60	64.00 ± 9.86	39.83 ± 21.71	41.00 ± 23.13	0.031	0.031
p-value ***	0.001		0.001			
**General health**	52.00 ± 9.24	72.00 ± 7.14	54.33 ± 22.07	53.00 ± 21.67	0.595	0.595
p-value ***	0.001		0.001			

*p-value- Before intervention

**p-value After intervention

***p-value Intragroup comparison

## Discussion

This study aimed to determine the role of a self-management program based on the 5 A nursing intervention model in the quality of life of hemodialysis patients. According to the findings, there were significant differences between the two groups after the intervention regarding the specific dimensions of life quality, such as cognitive performance, symptoms, sleeping status, dialysis, social support, and renal complications. Farbod et al. (2019) analyzed the effects of a 5 A self-management model on the quality of life of patients with hypertension. They reported that the mean scores of different areas of quality of life (physical, psychological, social, and environmental) and the total score of quality of life increased significantly in the intervention group as opposed to the control group after the intervention [15]. Khoshkhu et al. (2021) analyzed the effects of a 5 A-based program on self-care and quality of life among patients with hypertension. Their results indicated significant differences between the intervention and control groups after the intervention [16]. In a study entitled *Effects of 5 A Care Model on Quality of Life and Fatigue in Patients Undergoing Chemotherapy*, Zhang et al. (2021) indicated that the proposed model increased patients’ quality of life significantly and reduced their fatigue [17]. In another study entitled *Effects of 5 A-Based Self-Management Program on Fatigue and Dyspnea of Patients with Heart Failure*, Hajmohammadi et al. (2021) reported that the proposed model decreased fatigue and shortness of breath significantly among patients [14]. Hence, in line with the recent research findings, other studies have also found this model effective in certain factors, such as the quality of life and self-care among patients with chronic diseases. In this study, many dimensions of quality of life were improved significantly in the intervention group as opposed to the control group.

The total score of the general quality of life significantly increased among the patients in the intervention group; however, it significantly decreased in the control group. These findings indicated a significant difference between the two groups regarding the quality of life scores in all dimensions. In other words, most of the scores were improved in the intervention group; however, the scores declined in the control group. In fact, the intervention improved the general quality of life scores significantly in most dimensions. Ginanjar et al. (2017) used cell phones and the 5 A model to enhance the quality of life among patients with hypertension. According to their results, the scores of patients increased on all *SF–36* dimensions in the intervention group as opposed to the control group. They also reported significant differences in physical and emotional dimensions and pain [18].

In line with other studies, this survey indicated the favorable effects of the 5 A model on the quality of life of many patients with chronic conditions due to its person-based characteristics. The declined quality of life scores in the control group could be attributed to the specific sampling conditions. Many patients had constraints in attending the group sessions and doctor visits due to the public instructions during the COVID-19 pandemic, which could affect their quality of life.

Contrary to the survey by Javanoosh et al. (2016) examining the effect of the self-management program based on the 5 A model on the quality of life of the elderly with acute coronary syndrome, the results showed an increase in the average scores of all aspects of the quality of life in the intervention group, but, this increase was not statistically significant. This lack of significance can be due to the old age of the participants, as many factors are influential in their quality of life, and many functions and activities are reduced and declined per se due to the nature of acute coronary syndrome [19].

In addition to imposing high costs and excessive burdens on society due to side effects and dependence on health centers, hemodialysis can cause unfavorable changes to the quality of life. The first step in the 5 A model is based on a patient’s personal assessment, and each patient’s conditions and problems are considered in all steps. Hence, this model should be applied to hemodialysis patients more often. Moreover, non-attendance follow-ups via phone can help identify at-risk cases and prevent re-hospitalization or the emergence of risk factors in patients before the onset of a condition.

The researcher did not find any study investigating the effect of the self-management program based on the 5 A model on the quality of life of hemodialysis patients, and this study is one of the newest in this field. Therefore, we recommend using this model to improve the quality of life of dialysis patients. Also, we need more studies with larger samples.

This study faced certain limitations. For instance, there was only one dialysis center in the designated city; thus, the research team had to perform sampling in only one ward. Despite the iterated research emphasis, this limitation may have led to the distribution and sharing of information between the two groups, accounting for the insignificance of some dimensions. Moreover, many factors the researcher could not control may have affected the quality of life.

## Conclusion

The findings indicated that the 5 A nursing model improved the quality of life of hemodialysis patients. Many care services provided by healthcare centers, especially for patients with chronic conditions, are offered within a routine framework without considering each patient’s needs. For instance, some hemodialysis patients may have polyuria, whereas others may develop oliguria, requiring special care and instructions. These details are sometimes ignored. Therefore, self-management models such as the proposed one can improve these patients’ self-care and quality of life. However, the follow-up period was short in this study; therefore, it is recommended to prolong the follow-up period in subsequent studies.

## Data Availability

The datasets used during the current investigation are available from the corresponding author on reasonable request.
